# Musculocutaneous Pedicled Anterolateral Thigh Flap for Reconstruction of Stage IV Trochanteric Pressure Ulcers: Experience in Chronic and Acute Compression Injuries

**DOI:** 10.3390/jcm15051988

**Published:** 2026-03-05

**Authors:** Omer Kokacya, Ibrahim Tabakan, Gazi Kutalmis Yaprak, Ensari Yavuz, Erol Kesiktas

**Affiliations:** 1 Department of Plastic Reconstructive and Aesthetic Surgery, Faculty of Medicine, Cukurova University, Adana 01330, Türkiye; 2 Private Practice, İstanbul 34330, Türkiye

**Keywords:** pressure ulcer, trochanteric, anterolateral thigh flap, vastus lateralis, musculocutaneous flap

## Abstract

**Background/Objectives:** Trochanteric pressure ulcers represent a challenging reconstructive problem due to their depth, frequent infection, and tendency for recurrence. Durable coverage with well-vascularized tissue capable of effective dead-space management is essential for long-term stability. The pedicled musculocutaneous anterolateral thigh (ALT) flap offers substantial soft-tissue volume with reliable regional vascularity. **Methods:** A retrospective review was performed of consecutive patients with Stage IV trochanteric pressure ulcers who underwent reconstruction using musculocutaneous pedicled island ALT flaps between January 2020 and August 2023. Ulcers were classified according to the European Pressure Ulcer Advisory Panel, National Pressure Injury Advisory Panel, and Pan Pacific Pressure Injury Alliance International Guidelines. Patients with a minimum follow-up of 24 months were included. Demographic characteristics, ulcer etiology, prior flap history, comorbidities, flap dimensions, postoperative complications, and recurrence rates were analyzed. **Results:** Eight patients (4 males, 4 females; mean age 46.4 years, range 35–63) were included. Six ulcers (75%) were related to prolonged immobilization, and two (25%) developed following entrapment during the 2023 Kahramanmaraş earthquake. All donor sites were closed primarily. Minor recipient-site complications, including seroma and limited suture dehiscence, occurred in two cases. No partial or total flap necrosis was observed. During a mean follow-up of 42.4 months (minimum 24 months), no recurrence occurred. **Conclusions:** Musculocutaneous pedicled ALT flaps incorporating a substantial portion of the vastus lateralis muscle allowed effective dead-space obliteration and durable soft-tissue coverage in Stage IV trochanteric pressure ulcers. Primary donor-site closure was achievable without major morbidity. These findings support the use of the pedicled ALT flap as a consistent regional option in complex trochanteric defects, including both chronic immobilization-related and acute compression-related cases.

## 1. Introduction

Trochanteric pressure ulcers represent a challenging subset of chronic wounds, most frequently encountered in patients with prolonged immobilization due to paraplegia, spinal cord injury, or severe neurological disorders. The greater trochanter is particularly susceptible to sustained pressure injury because of its prominent osseous anatomy and relatively limited overlying soft tissue coverage. Persistent compression, shear stress, and ischemia may lead to progressive tissue breakdown extending to the trochanteric bursa and femoral periosteum, ultimately resulting in deep Stage IV defects that require surgical reconstruction for durable closure rather than conservative wound care alone. Recent reports continue to highlight the complexity of pressure ulcer reconstruction and the need for individualized surgical planning in advanced defects [1,2].

The cornerstone of management in Stage IV trochanteric pressure ulcers is radical debridement followed by reconstruction with well-vascularized tissue capable of obliterating dead space and providing durable coverage. The tensor fascia lata (TFL) musculocutaneous flap remains a well-established regional option for trochanteric reconstruction [3], and posterior thigh flaps have also been described, including modified designs aimed at improving reach and donor-site outcomes [4].

In recent years, the pedicled anterolateral thigh (ALT) flap has been increasingly utilized as a reconstructive alternative for trochanteric defects [5,6]. Owing to its reliable vascular anatomy and the length of the descending branch of the lateral circumflex femoral artery, the pedicled ALT flap provides a wide arc of rotation, allowing coverage of defects in the groin, lower abdominal, gluteal, perineal, and thigh areas [7,8]. Incorporation of the vastus lateralis muscle allows additional tissue bulk for dead-space obliteration in extensive defects, while the length and reliability of its vascular pedicle facilitate regional transposition to the trochanteric area without the need for microsurgical anastomosis [5,6].

Although chronic immobilization remains the predominant cause of trochanteric pressure ulcers, pressure-induced soft tissue necrosis may also occur in acute settings, including disaster-related compression injuries. Prolonged entrapment during earthquakes can lead to crush-associated ischemic damage and traumatic rhabdomyolysis, resulting in both local tissue necrosis and systemic complications [9,10]. Unlike chronic pressure ulcers—where long-standing muscle atrophy and fibrosis are common—acute compression injuries typically affect previously healthy tissues. After debridement, these defects may therefore present with substantial volume loss but relatively preserved surrounding tissue quality, which may influence reconstructive planning.

In this retrospective single-center study, we present our experience using musculocutaneous pedicled island ALT flaps incorporating the entire vastus lateralis muscle for the reconstruction of Stage IV trochanteric pressure ulcers. The study includes cases related to chronic immobilization as well as acute compression injuries observed after the 2023 Kahramanmaraş earthquake. Surgical outcomes are analyzed with long-term follow-up.

## 2. Materials and Methods

In this retrospective study, we reviewed all consecutive patients with Stage IV trochanteric pressure ulcers, classified according to the European Pressure Ulcer Advisory Panel, National Pressure Injury Advisory Panel, and Pan Pacific Pressure Injury Alliance international guidelines [11], who underwent reconstruction with musculocutaneous pedicled island ALT flaps at our institution between January 2020 and August 2023.

Patients with a minimum follow-up of 24 months were included. Medical records were retrospectively reviewed, and the following variables were analyzed: demographic characteristics, ulcer etiology, history of prior flap reconstruction, comorbidities, defect size, flap dimensions, postoperative complications, and recurrence rates.

Postoperative complications were defined as any flap-related or donor-site adverse events occurring during the follow-up period, including partial or total flap necrosis, wound dehiscence, seroma, infection, and the need for revision surgery. Complications and recurrence were assessed through routine outpatient follow-up visits based on clinical examination.

### Operative Technique

Flap design was performed along a line drawn between the anterior superior iliac spine and the superolateral border of the patella. The midpoint of this line was identified, and perforators were located using a handheld Doppler probe within a 3 cm radius. The skin paddle was designed according to the dimensions of the trochanteric defect and intentionally marked slightly larger to allow adequate bulk and tension-free inset. When necessary, excess portions of the skin paddle were deepithelialized and buried within the defect to assist in dead-space obliteration.

A medial incision was made first, extending to the deep fascia over the rectus femoris muscle. Subfascial dissection proceeded laterally toward the intermuscular plane between the rectus femoris and vastus lateralis muscles, and the perforator was identified. The perforator was unroofed and traced proximally to confirm its continuity with the descending branch of the lateral circumflex femoral artery (LCFA). Limited intramuscular dissection was performed as needed to visualize the pedicle and obtain adequate mobility.

All flaps were elevated as musculocutaneous flaps incorporating a substantial portion of the vastus lateralis muscle to provide sufficient vascularized bulk for reconstruction of Stage IV defects. The descending branch of the LCFA was dissected proximally to achieve adequate pedicle length. The pivot point was located at the proximal portion of the descending branch, allowing rotation toward the trochanteric region without tension.

The skin bridge between the donor site and the defect was divided, and the flap was transposed directly to the trochanteric area. The pedicle was positioned carefully to avoid tension or torsion. Care was taken to preserve the remaining branches of the LCFA in order to maintain the vascular integrity of the tensor fascia lata region for potential future reconstruction.

The donor site was closed primarily in all patients.

## 3. Results

Demographic characteristics, ulcer etiology, comorbidities, history of prior flap surgery, defect and flap dimensions, surgical outcomes, and follow-up data are summarized in Table 1.

A total of eight patients were included in the study, with a mean age of 46.4 years (range, 35–63 years). The cohort consisted of four female (50%) and four male (50%) patients. Comorbidities were present in three patients (37.5%), while five patients (62.5%) had no significant comorbid conditions. Six patients (75%) developed trochanteric pressure ulcers as a result of long-term immobilization, whereas two patients (25%) developed ulcers following entrapment under rubble during the 2023 Kahramanmaraş earthquake. Three patients (37.5%) had undergone at least one prior flap reconstruction of the trochanteric region.

Flap size ranged from 28 cm × 12 cm (336 cm^2^) to 35 cm × 16 cm (560 cm^2^). All donor sites were closed primarily. No partial or total flap necrosis occurred. Minor complications were observed in two patients, consisting of seroma and suture dehiscence at the recipient site. The mean follow-up duration was 42.4 months, with a minimum follow-up of 24 months. No recurrence was observed in any patient during the follow-up period.

### 3.1. Case Presentations

#### 3.1.1. Patient 1

**Figure 1:**
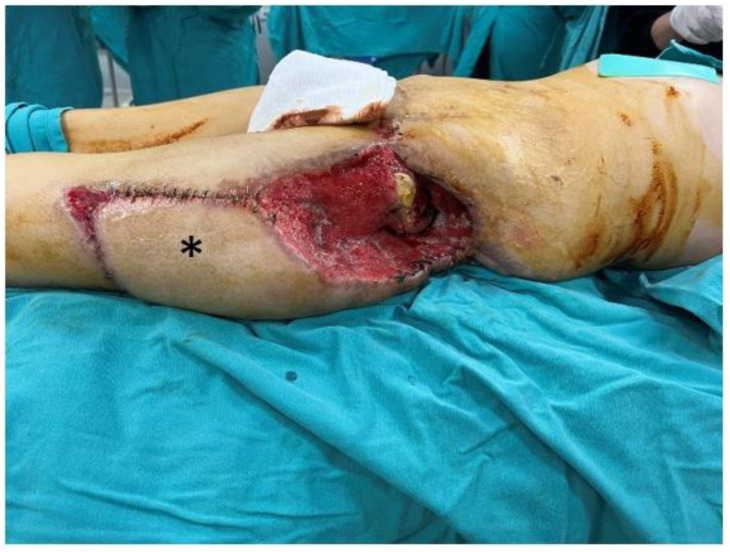
A 24 cm × 16 cm trochanteric defect with exposure of the femoral head. The asterisk (*) denotes the posterior thigh flap that was returned to its original donor site.

**Figure 2:**
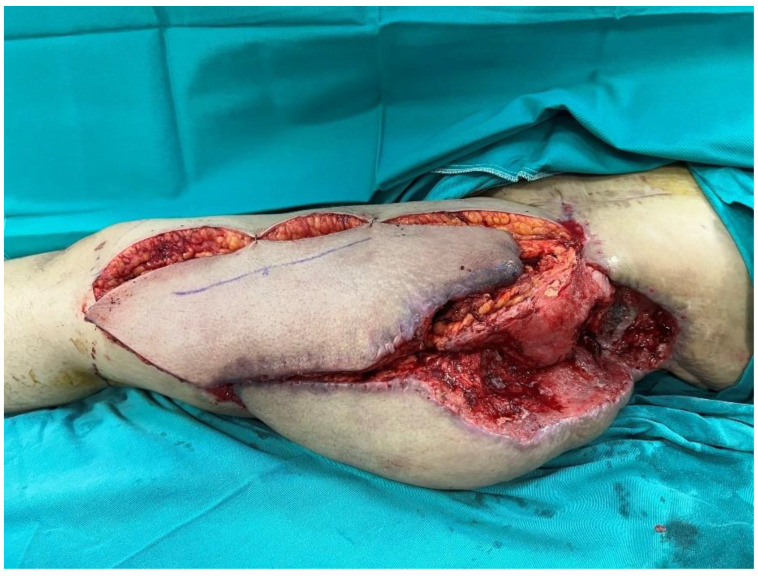
Musculocutaneous anterolateral thigh (ALT) flap with a 35 cm × 16 cm skin paddle.

**Figure 3:**
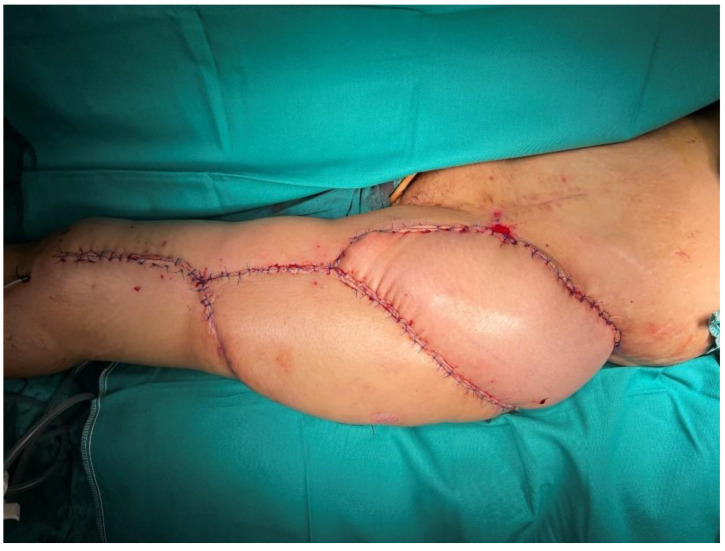
The donor site was closed primarily by medial advancement of the posterior thigh flap reconstructed at the referring institution.

A 40-year-old female patient developed a trochanteric pressure ulcer on the left hip after being trapped under rubble during the 2023 Kahramanmaraş earthquake. At another center, the defect was initially managed with serial debridements, wound care, and reconstruction using a posterior thigh flap. However, due to poor flap adaptation and persistent discharge beneath the flap, the flap was returned to its donor site, and the patient was referred to our institution. On presentation, a 24 cm × 16 cm defect was observed in the left trochanteric region with exposure of the femoral head (Figure 1). Handheld Doppler assessment confirmed intact perforators suitable for an ALT flap. A 35 cm × 16 cm musculocutaneous ALT flap, incorporating a substantial portion of the vastus lateralis muscle, was elevated (Figure 2). The flap was inset into the defect, and the donor site was closed primarily by medial advancement of the previously used posterior thigh flap (Figure 3).

#### 3.1.2. Patient 2

**Figure 4:**
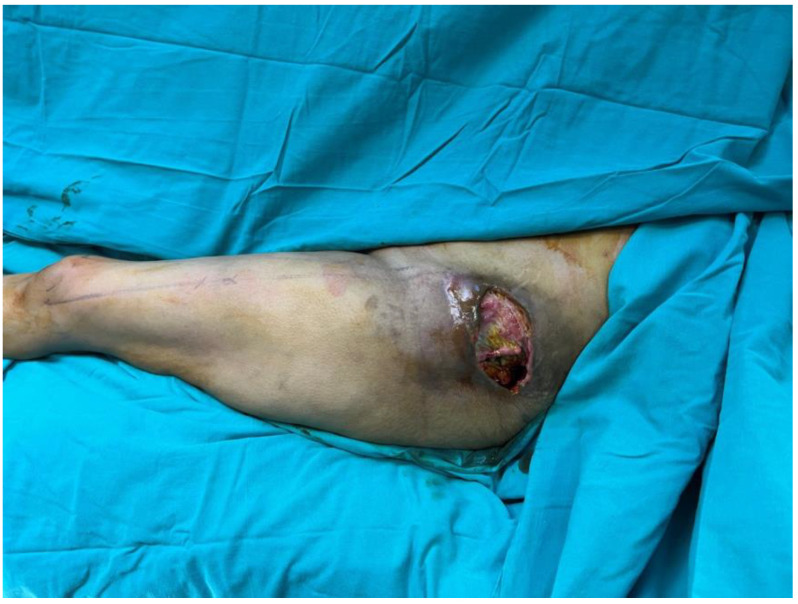
Clinical presentation of the trochanteric pressure ulcer prior to surgical debridement.

**Figure 5:**
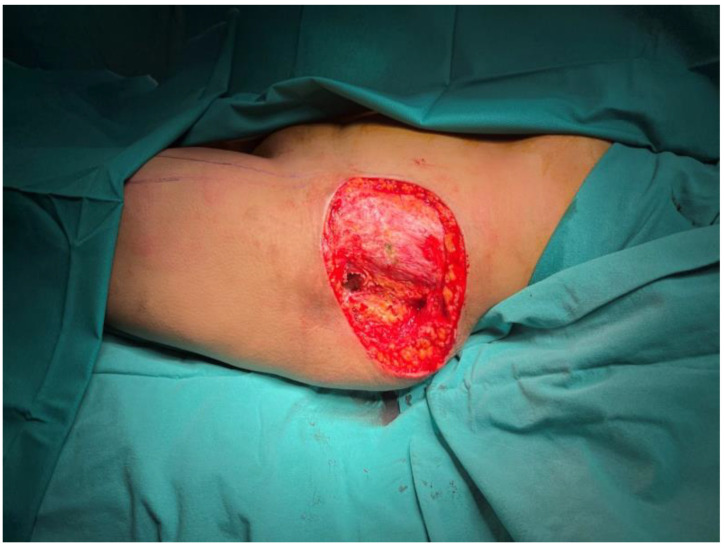
The trochanteric defect following surgical debridement.

**Figure 6:**
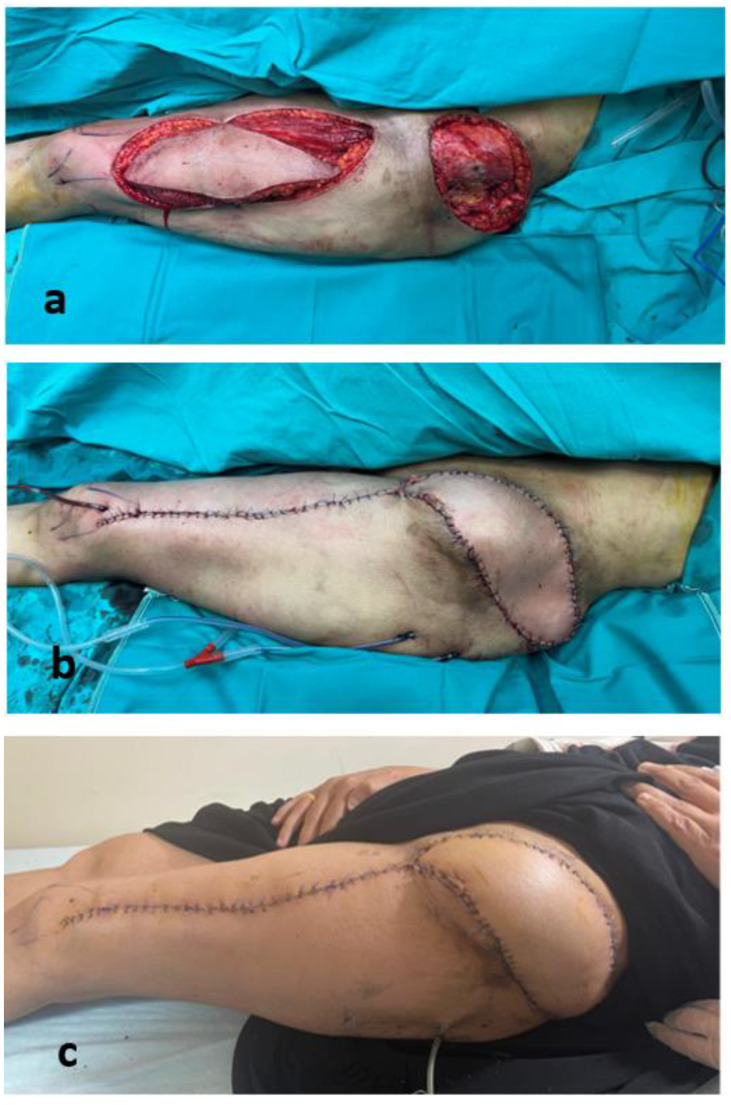
Intraoperative and early postoperative views: (**a**) before flap inset, (**b**) after flap inset, and (**c**) early postoperative appearance.

A 41-year-old female patient was rescued from under the rubble following the 2023 Kahramanmaraş earthquake. Her initial emergency management and follow-up care were performed at another center. Six weeks later, she presented to our outpatient clinic with a non-healing open wound in the left trochanteric region (Figure 4), and debridement with simultaneous reconstruction was planned. Following radical debridement of necrotic tissue, a 12 cm × 18 cm defect with exposure of the trochanteric bursa was identified (Figure 5). The defect was reconstructed using a 35 cm × 14 cm musculocutaneous anterolateral thigh (ALT) flap. The donor site was closed primarily. The postoperative course was uneventful, and complete wound healing was achieved (Figure 6).

#### 3.1.3. Patient 7

**Figure 7:**
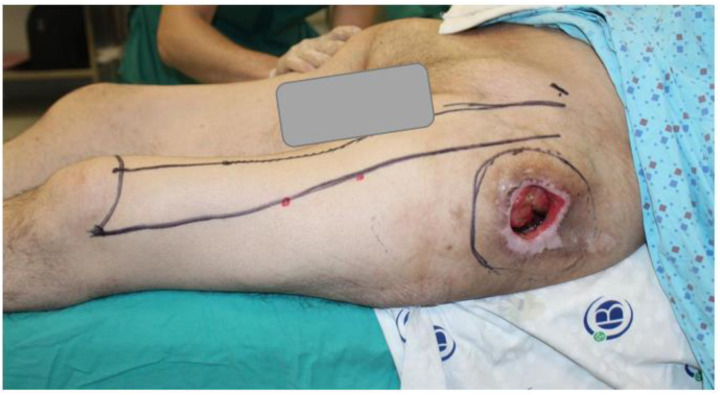
Preoperative view showing surgical markings for debridement and flap design.

**Figure 8:**
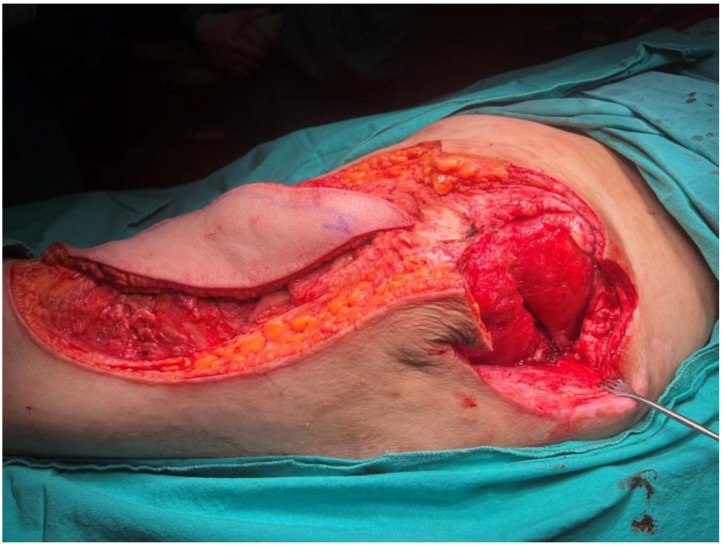
Intraoperative view demonstrating the debrided defect and the harvested musculocutaneous ALT flap.

**Figure 9:**
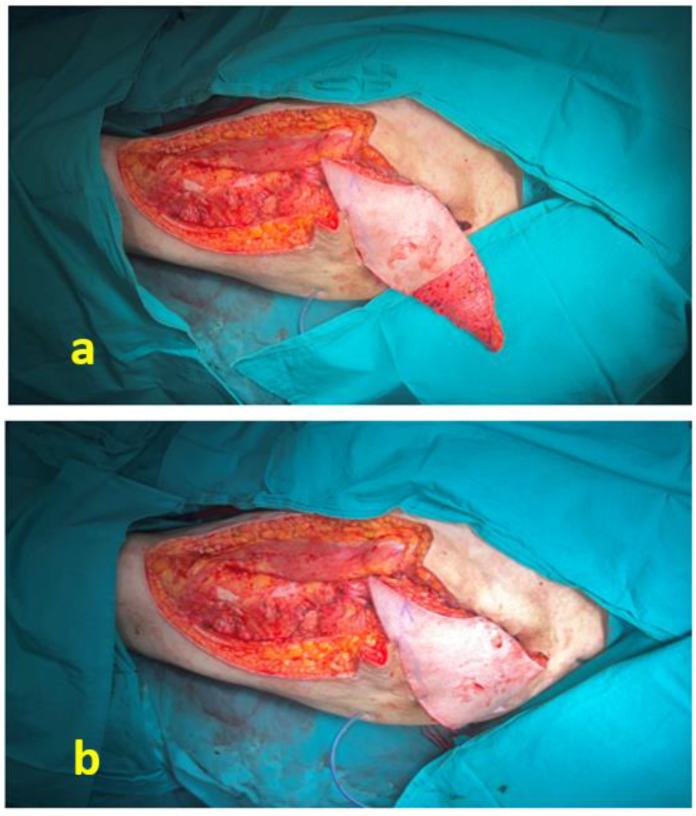
(**a**) The distal one-third of the flap was deepithelialized. (**b**) The deepithelialized portion of the flap was buried to obliterate the dead space.

**Figure 10:**
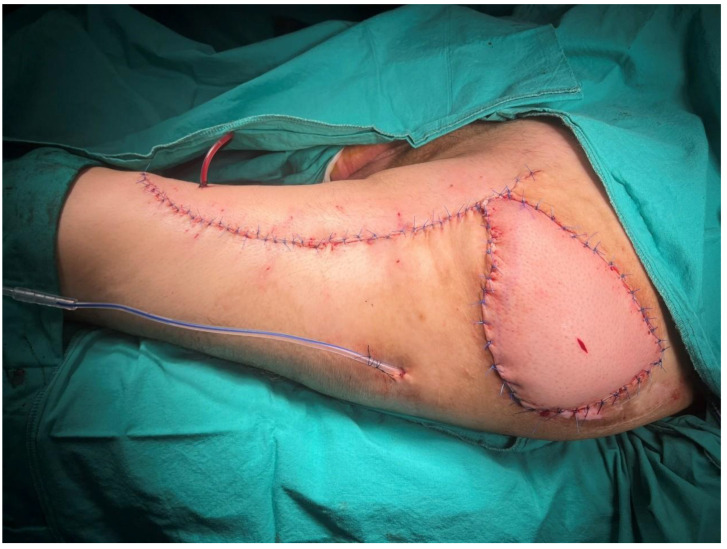
Final intraoperative view following flap inset.

A 48-year-old male patient had been paraplegic for 5 years following spinal surgery. He had previously undergone surgical treatment for a sacral pressure ulcer and was admitted for reconstruction of a trochanteric pressure ulcer (Figure 7). After radical surgical debridement, a 12 cm × 12 cm defect was identified. A musculocutaneous anterolateral thigh (ALT) flap with a 28 cm × 12 cm skin paddle was elevated (Figure 8). In addition to the included vastus lateralis muscle, the distal deepithelialized portion of the flap was used to obliterate the dead space (Figure 9). The donor site was closed primarily (Figure 10).

## 4. Discussion

Thorough surgical debridement followed by reconstruction with well-vascularized tissue remains the cornerstone of treatment in advanced trochanteric pressure ulcers [11]. These defects are particularly challenging because of their depth, frequent association with infection, and propensity for recurrence. Tissue loss may extend to the trochanteric bursa and femoral periosteum, and in selected cases with refractory infection or extensive bone involvement, more aggressive surgical strategies may be required to achieve durable control. In such circumstances, reconstruction with vascularized muscle-containing flaps is essential for dead-space obliteration and infection control [12].

Since the original description of the tensor fascia lata (TFL) musculocutaneous flap by Nahai et al. [3], the TFL flap has been widely accepted as a reliable regional option for trochanteric reconstruction. Various modifications have been described to improve reach and minimize donor-site morbidity [13,14]. Additionally, posterior thigh flap modifications can be used for pressure sores in this region [4].

The anterolateral thigh (ALT) flap has progressively gained acceptance in both free and pedicled forms. As a free flap, the ALT flap has been applied across a wide range of reconstructive indications [15], including high-risk diabetic foot reconstruction [16], complex orthopedic trauma requiring stable soft tissue coverage [17], and limb salvage in severe frostbite-related tissue loss [18]. In recent years, the pedicled anterolateral thigh (ALT) flap has emerged as a versatile regional alternative for complex trochanteric defects [5,6]. Owing to its reliable vascular anatomy and the length of the descending branch of the lateral circumflex femoral artery, the pedicled ALT flap provides a wide arc of rotation, allowing coverage of defects in the groin, lower abdominal, gluteal, perineal, and thigh regions [7,8]. Tzeng et al. [19] reported proximally pedicled ALT flaps for trochanteric reconstruction, while Wang et al. [20] described musculocutaneous pedicled ALT flaps incorporating a portion of the vastus lateralis muscle without complete perforator skeletonization, emphasizing technical simplicity and vascular safety. Incorporation of the vastus lateralis muscle allows additional tissue bulk for dead-space obliteration in extensive defects, while the length and reliability of its vascular pedicle facilitate regional transposition to the trochanteric area without the need for microsurgical anastomosis.

In our series, all defects were reconstructed using musculocutaneous pedicled ALT flaps incorporating a substantial portion of the vastus lateralis muscle. This approach provided sufficient vascularized bulk to effectively obliterate deep cavities following radical debridement. Also, desepitalization of the flap further improves the dead space reduction. Limited perforator dissection was performed without complete skeletonization, simplifying flap harvest while preserving vascular reliability.

Despite the relatively large skin paddles, all donor sites were closed primarily without donor-site complications. Primary closure was generally achievable because the flap was harvested as a musculocutaneous unit, which helped reduce lateral thigh bulk and made tissue advancement easier. Although local tissue characteristics differed between chronic immobilization-related ulcers and acute compression-related defects—chronic cases often demonstrating varying degrees of muscle atrophy and scarring, and acute cases generally presenting with relatively preserved soft tissues—these differences did not appear to affect donor-site closure in this series.

An important aspect of this study is the inclusion of patients who developed acute compression-related necrosis following the 2023 Kahramanmaraş earthquake. In contrast to chronic pressure ulcers associated with prolonged immobilization and progressive muscle atrophy [21], acute compression injuries may involve previously healthy tissues prior to ischemic insult [9]. After debridement, these cases may present with significant volume loss and relatively preserved surrounding tissue quality. In this setting, the ALT flap offers distinct advantages by providing generous vascularized muscle bulk and a reliable pedicled blood supply without the need for microsurgical anastomosis. The favorable outcomes observed in both chronic immobilization-related ulcers and earthquake-related acute compression injuries in our cohort suggest that this technique can be applied across different pathophysiological settings.

More extensive musculocutaneous reconstructions incorporating additional muscle components have been described for highly complex trochanteric and ischial defects, particularly in association with femoral head resection or advanced bone involvement [22,23]. Although such approaches remain valuable in selected cases, all defects in our series were successfully managed using the pedicled musculocutaneous ALT flap alone, without the need for additional muscle harvest.

In our current clinical practice, flap selection is guided primarily by defect depth, cavity size, and tissue quality. For non-Stage IV trochanteric pressure ulcers without deep cavitary involvement, regional options such as the tensor fascia lata (TFL) flap or local rotation or hatchet flaps from the proximal thigh or gluteal region are generally preferred. In contrast, for Stage IV defects characterized by deep cavities and significant tissue loss, a musculocutaneous pedicled ALT flap is selected to provide adequate vascularized muscle bulk for dead-space obliteration and durable coverage. Li et al. reported that, compared with the TFL flap, the pedicled ALT flap can provide relatively greater muscle volume and a larger skin paddle, facilitating obliteration of deep cavities and tension-free reconstruction in extensive trochanteric pressure sores [24]. Our institutional algorithm similarly favors musculocutaneous pedicled ALT flaps in deep Stage IV defects requiring substantial dead-space management.

This study has several limitations. It represents a retrospective single-center experience with a limited number of patients and without a comparative control group. Only one reconstructive technique was evaluated. Therefore, the findings should be interpreted as an institutional experience rather than definitive evidence of superiority over other established reconstructive options. This study presents a technical application of an established musculocutaneous pedicled ALT flap within a defined, defect-oriented clinical algorithm. The results should be understood in this context as an illustration of practical feasibility and long-term outcomes in both chronic and acute compression-related defects. Larger comparative studies are needed to further clarify the role of pedicled ALT flaps in trochanteric pressure ulcer reconstruction.

## 5. Conclusions

Musculocutaneous pedicled anterolateral thigh flaps represent a reliable and adaptable option for reconstruction of deep Stage IV trochanteric pressure ulcers requiring effective dead-space management and durable soft-tissue coverage. In our experience, the incorporation of a substantial portion of the vastus lateralis muscle allowed adequate cavity obliteration while permitting primary donor-site closure without major morbidity. Favorable outcomes were observed in both chronic immobilization-related ulcers and acute compression-related defects, suggesting that this technique can be applied across different clinical scenarios encountered in trochanteric reconstruction. Further comparative studies are warranted to better define its long-term performance relative to other established regional flaps.

## Figures and Tables

**Table 1:** Demographic characteristics, ulcer etiology, comorbidities, prior flap reconstruction, defect and flap dimensions, and surgical outcomes.

	Age	Sex	Etiology	Comorbidities	Prior Flap Reconstruction	Defect Size (cm)	Flap Size (cm)	Donor Site Closure	Complications	Follow-Up (Months)	Recurrence
1	40	Female	2023 Kahramanmaraş earthquake-related compression injury	None	PTF	24 × 16	35 × 16	Primary	None	26	No
2	41	Female	2023 Kahramanmaraş earthquake-related compression injury	None	None	12 × 18	35 × 14	Primary	None	27	No
3	35	Female	Quadriplegia for 4 years secondary to hydrocephalus surgery	Epilepsy, mental and motor retardation, scoliosis	None	16 × 14	30 × 15	Primary	None	24 **	No
4	58	Male	Paraplegia for 12 years secondary to a traffic accident	Diabetes mellitus, coronary artery disease	TFL, PTF	12 × 15	30 × 14	Primary	Seroma	36	No
5	41	Male	Paraplegia for 11 years secondary to a traffic accident	None	TFL, local fasciocutaneous flap	16 × 14	32 × 14	Primary	Suture dehiscence	49	No
6	63	Female	Immobilization due to muscle weakness for 4 years	Hypertension, diabetes mellitus	None	15 × 10	28 × 12	Primary	None	53	No
7	48	Male	Paraplegia for 5 years secondary to spinal surgery	None	None	12 × 12	28 × 12	Primary	None	56	No
8	45	Male	Paraplegia for 8 years secondary to a fall from height	None	None	14 × 12	32 × 12	Primary	None	58	No

Abbreviations: TFL, tensor fascia lata flap; PTF, posterior thigh flap. ** Patient died due to causes unrelated to surgery.

## Data Availability

The original contributions presented in this study are included in the article. Further inquiries can be directed to the corresponding author.

## Abbreviations

The following abbreviations are used in this manuscript:

ALT	Anterolateral thigh flap
TFL	Tensor fascia lata flap
PTF	Posterior thigh flap
LCFA	Lateral circumflex femoral artery
